# Neural activity in the human anterior thalamus during natural vision

**DOI:** 10.1038/s41598-021-96588-x

**Published:** 2021-09-01

**Authors:** Marcin Leszczynski, Leila Chaieb, Tobias Staudigl, Simon Jonas Enkirch, Juergen Fell, Charles E. Schroeder

**Affiliations:** 1 Department of Psychiatry, College of Physicians and Surgeons, Columbia University Medical Center, 1051 Riverside Drive Kolb Annex Rm 561, New York, NY 10032 USA; 2 Translational Neuroscience Division, Center for Biomedical Imaging and Neuromodulation, Nathan Kline Institute, Orangeburg, NY USA; 3 Department of Epileptology, University Hospital Bonn, Bonn, Germany; 4 Department of Psychology, Ludwig-Maximilians-Universität München, Munich, Germany; 5 Department of Neuroradiology, University Hospital Bonn, Bonn, Germany

**Keywords:** Sensory processing, Neurophysiology, Attention

## Abstract

In natural vision humans and other primates explore environment by *active sensing*, using saccadic eye movements to relocate the fovea and sample different bits of information multiple times per second. Saccades induce a phase reset of ongoing neuronal oscillations in primary and higher-order visual cortices and in the medial temporal lobe. As a result, neuron ensembles are shifted to a common state at the time visual input propagates through the system (i.e., just after fixation). The extent of the brain’s circuitry that is modulated by saccades is not yet known. Here, we evaluate the possibility that saccadic phase reset impacts the anterior nuclei of the thalamus (ANT). Using recordings in the human thalamus of three surgical patients during natural vision, we found that saccades and visual stimulus onset both modulate neural activity, but with distinct field potential morphologies. Specifically, we found that fixation-locked field potentials had a component that preceded saccade onset. It was followed by an early negativity around 50 ms after fixation onset which is significantly faster than any response to visual stimulus presentation. The timing of these events suggests that the ANT is *predictively* modulated before the saccadic eye movement. We also found oscillatory phase concentration, peaking at 3–4 Hz, coincident with suppression of Broadband High-frequency Activity (BHA; 80–180 Hz), both locked to fixation onset supporting the idea that neural oscillations in these nuclei are reorganized to a low excitability state right after fixation onset. These findings show that during real-world natural visual exploration neural dynamics in the human ANT is influenced by visual and oculomotor events, which supports the idea that ANT, apart from their contribution to episodic memory, also play a role in natural vision.

## Introduction

Both human and non-human primates sample visual scenes actively by systematically shifting eye gaze several times per second^1,2^. These eye movements modulate neural activity along the visual pathway from the lateral geniculate nucleus^3–7^ through ascending stages of visual cortex^8–15^ up to the medial temporal lobe (MTL) including the hippocampus and entorhinal cortex^16–24^ (for review see^25^).

**Figure 1:**
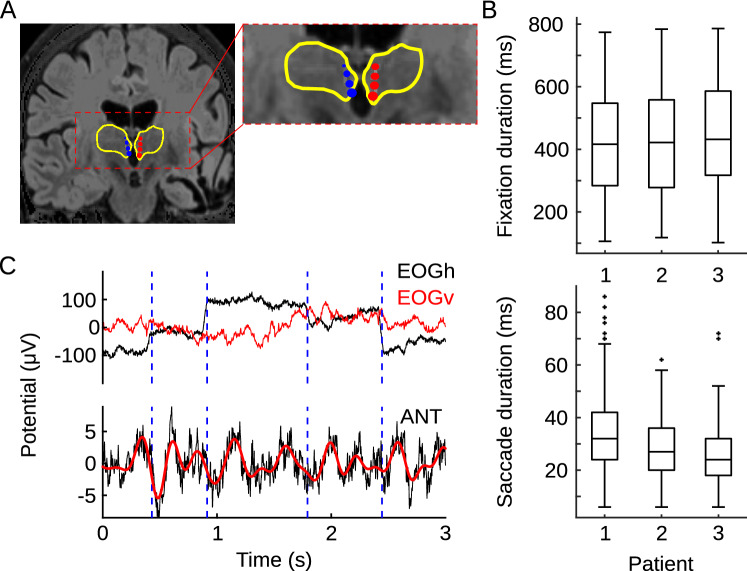
Electrode locations, example field potential trace and behavior. (**A**) Bilateral leads implanted into the anterior nuclei of the thalamus marked with blue (right ANT) and red (left ANT) in a single patient (4 contacts per side). Inter-contact space of 3 mm (contact height of 1.5 mm with 1.5 mm spacing). Yellow contours depict boundaries of the thalamus. (**B**) Distribution of fixation (upper panel) and saccade (lower panel) durations for three patients. Box plots indicate 25th, median and 75th percentile, whiskers extend to extreme values not considered outliers while outliers are marked with crosses. Because we used the EOG signal to define time points of fixation and saccade onsets, it is possible that smaller eye movements were not detected (see Limitations). (**C**) An example of 3 s long recording trace with horizontal (EOGh; black trace) and vertical (EOGv; red trace) part of EOG signal (upper panel). Horizontal dashed lines indicate fixation onset time points. The same interval of 3 s field potentials from an example site in the ANT. Black trace presents raw signal; red trace shows signal filtered in 2–5 Hz (lower panel).

Earlier studies of the effects of saccades in the dark^3,5,6,9,26–29^, and more recent studies that minimize saccade-related visual input by various means^7,15^ confirm the proposition that nonretinal “corollary discharge” (CD) signals generated in parallel to saccades, modulate the excitability of neurons in visual pathways structures^30^. For example, Barczak et al. showed that nonretinal saccadic signals reset ongoing excitability fluctuations (oscillations) in V1 neuron ensembles to a high excitability phase, and that this effect amplifies their response to visual inputs arriving immediately after the saccade^15^ (i.e., at fixation onset). This phase modulation is primarily observed in theta and alpha ranges—key physiological signatures of active sensing^25,31–33^. Areas displaying saccade-dependent modulations of neural activity include higher order regions like the MTL suggesting that the network which is influenced by eye movements is broader than previously assumed. However, aside from the tectal nuclei^30,34,35^ and the lateral geniculate nucleus of the thalamus^3–7,28^, the degree to which saccade-related signals impact other subcortical structures has not been systematically investigated. In particular, it is unknown whether non-visual nuclei of the thalamus are also synchronized to the rhythm of eye movements during natural active vision. Because saccadic eye movements influence neural activity in the hippocampus and the hippocampus modulates activity in the ANT through both direct and indirect connections^36^, we hypothesized that the ANT might also synchronize to the rhythm of saccadic exploration. Such synchronized activity could enhance both local neural processing but also network interactions^37^ within the hippocampal-diencephalic system. Since the ANT has been suggested to play a role in mnemonic^38–40^ rather the sensory processes, demonstrating that neural activity in the ANT is modulated during natural vision would add a new dimension to our thinking about the ANT function. To this end, we leveraged a unique dataset: intracranial electroencephalography (iEEG) recordings from ANT in patients implanted for deep brain stimulation treatment of epilepsy^41^ (Fig. 1A).

Based on the interconnectivity of the ANT with other brain regions like the hippocampus and anterior cingulate cortex^42,43^ as well as their apparent receipt of direct retinal afferents^44^ we hypothesized that neuronal activity in the ANT﻿﻿ might also synchronize to the rhythm of saccadic eye movements. We tested three specific predictions: (1) Saccades perturb ongoing field potentials in the human ANT; (2) The phase of low frequency oscillations clusters at or just after fixation onset as measured by the inter-trial phase coherence (ITC); (3) BHA, a measure of neuronal processes correlated but separable from neuronal spiking^45–49^, is systematically modulated by the saccade-fixation cycle—an increase would suggest reset to a high—while a decrease would suggest reset to a low-excitability phase. Our findings provide support for all three hypotheses. We observed that the neural activity in the human ANT is modulated by both visual and possibly also non-retinal elements. These findings advance our understanding of the ANT operations suggesting that apart from a role in episodic memory, they also play a role in vision.

## Results

### Fixation-locked field potentials in human ANT

Using frontal scalp electroencephalographic (EEG) and electro-oculogram (EOG) electrodes we detected time points of saccade and fixation onset (Fig. 1B, C; see “Methods” section). This is possible because eye movements generate large magnitude electric field fluctuations (i.e., EOG) that are measurable at scalp EEG across multiple frontal channels^50^. Combining simultaneous EEG and direct ANT recordings we could thus study the impact of eye movements (as detected from EEG/EOG) on neural activity in human anterior nuclei of the thalamus (Fig. 1).

**Figure 2:**
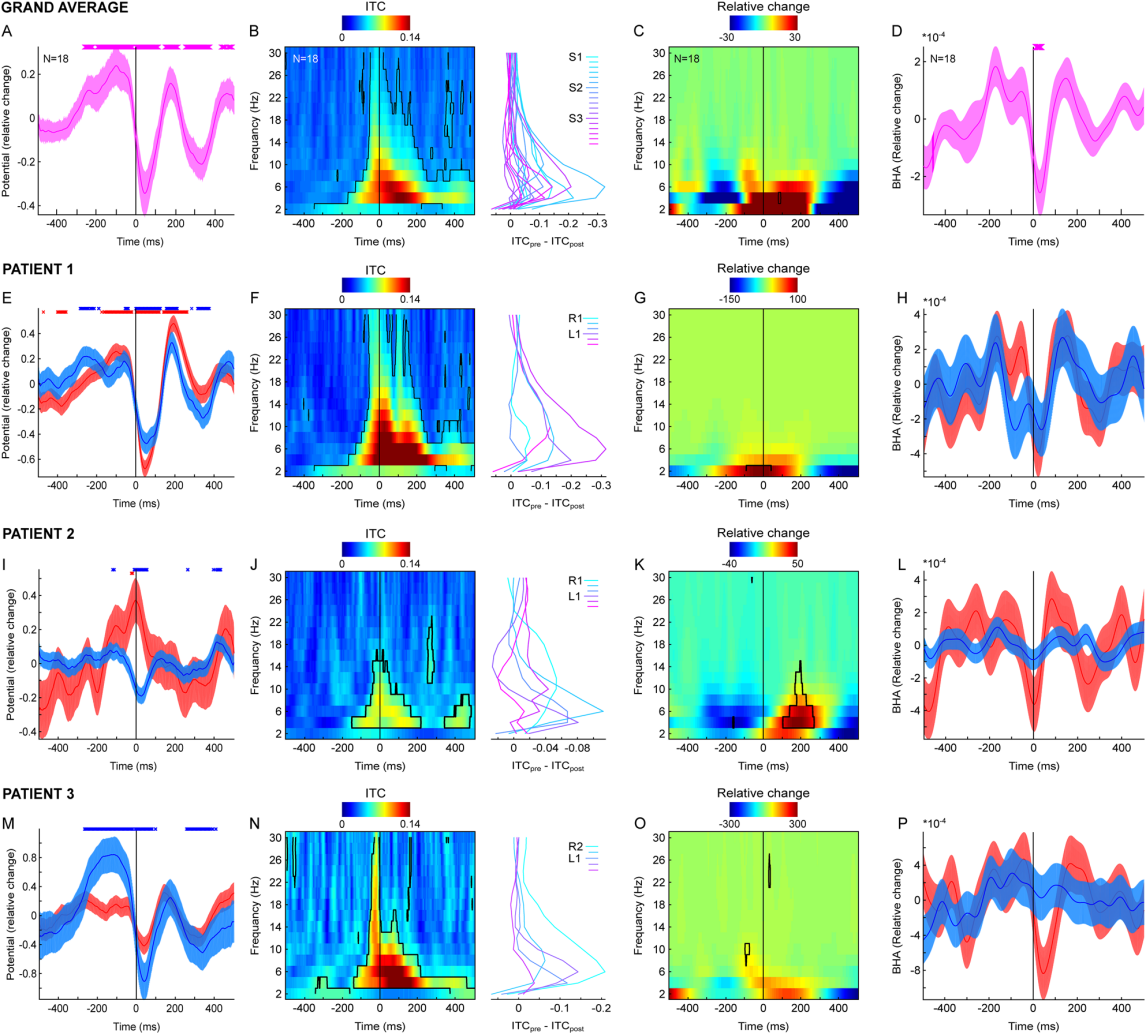
Fixation-locked neural activity in human ANT. (**A**) Grand average fixation-locked field potentials (N = 18 ANT channels, 3 patients). Vertical lines show the point of fixation onset. Markers above indicate significant time points (*p* < 0.05; permutation test). (**B**) Color map shows ITC (time on x-axis, frequency on y-axis). Contours depict significant time–frequency points (permutation test *p* < 0.05). Vertical black line indicates fixation onset. Line plots on the right side indicate ITC change from pre- (− 300: − 100 ms) to post-fixation (0:100 ms) time window separately for each channel. (**C**) Color map shows fixation locked power relative to trial average (time on x-axis, frequency on y-axis). Contours present change from surrogate distribution (permutation test *p* < 0.05). (**D**) Fixation-locked BHA (80–180 Hz). Vertical black line indicates fixation onset. Markers above indicate significant time points (permutation test *p* < 0.05). (**E**) Fixation-locked field potentials (patient 1) from channels on the right ANT (blue) and left ANT (red). Vertical black lines show the point of fixation onset. Markers above indicate significant time points (permutation test *p* < 0.05). (**F**) Same as B but for data from patient 1. (**G**) Same as C but for data from patient 1. (**H**) Fixation-locked BHA (patient 1) from channels on the right ANT (blue) and left ANT (red). Vertical black lines show the point of fixation onset. Data from patient 2 (**I**–**L**) and patient 3 (**M**–**P**) plotted with the same convention as (**E**–**H**). Note that the reversed polarity in patient 2 left channels might be explained by a more posterior and dorsal trajectory of the left electrode shaft. Furthermore, patient 3 had the right side electrode placed deep with ventral channels showing no signal (note only R2 and R3 are presented; see “Methods” section for more details). All results are controlled for multiple comparisons with Benjamini and Hochberg/Yekutieli false discovery rate procedure. Shading reflects standard error of the mean (SEM).

As in our earlier studies^9,15,49^, we focus on neural events related to the end of the saccade (fixation onset) as this is an event with clear perceptual relevance; i.e., the point at which retinal inflow begins in the ascending visual pathways. First, we tested whether fixation onset-related field potentials (FPs) are modulated relative to a surrogate distribution. The surrogates (N = 1000) were created by locking intact segments of ANT field potentials to pseudo-fixations (i.e., random time points uniformly distributed across the entire recording session; see “Methods” section). We observed that the magnitude of FPs (averaged across all ANT contacts in 3 patients) departs from the surrogate distribution at multiple time points (permutation test; *p* < 0.05; FDR controlled for multiple comparisons; see “Methods” section). An initial positive deflection started 260 ms before fixation onset and peaked around 100 ms before fixation onset. It was followed by a negative deflection peaking at 40–50 ms after fixation onset and another positive deflection peaking around 160 ms after fixation onset (Fig. 2A). We also observed that FPs exceeded the surrogate distribution in each of the three participants (see Fig. 2E, I, M). The earliest ERP deflection substantially precedes saccadic eye movement, suggesting that activity in the ANT is influenced not only by motion stimulation of the retina, but also by non-retinal signals. Next, we investigated how the saccade-fixation cycle influences spectral phase and power in the ANT. To this end, we studied fixation-locked ITC, power spectra across a range of low frequencies (1–30 Hz) and BHA power (80–180 Hz; see “Methods” section). Overall, we found that ITC increased above the 95th surrogate percentile (corresponding to *p* < 0.05; FDR controlled for multiple comparisons) across multiple time and frequency points with strongest effects of phase clustering at 3–4 Hz around 120 ms after fixation onset (see Fig. 2B). This effect was evident in each of the three patients (see Fig. 2F, J, N). In contrast we detected weak modulations of power relative to their surrogate distributions (Fig. 2C, G, K, O). We found a small fixation-locked BHA decrease around 40–50 ms after fixation onset (Fig. 2D). While the effect of BHA did not exceed single subject surrogate distribution it showed a consistent direction with the dip in the BHA observed right after fixation onset for each of the patients (Fig. 2H, L, P). Note that while all three effects (i.e., BHA drop after fixation onset, increased ITC and ERP modulation) are consistent across subjects, polarity of the ERP is reversed in the patient's 2 left electrode shaft. Careful inspection of single channel ERPs in this patient revealed that while all three bipolar channels on the right shaft showed a negative going deflection consistent with the group results, two deepest contacts on the left shaft (ANTL1 and ANTL2) showed a positive deflection of the ERP and the most superficial channel (i.e., bipolar ANTL3) had no detectable ERP around the time of fixation onset. This difference between left and right shaft in patient 2 might be explained by a more posterior and inferior placement of the left ANT electrode compared to that on the right side (see also^41^). One possibility is that channels on the left side are picking up signals from another nuclei (e.g., medial dorsal nucleus).

These results show that during natural vision the ANT field potentials are synchronized to the rhythm of eye movements. There are several processes which might contribute to these effects. At each fixation a volley of visual input is initiated in the retina and this information is then processed by a succession of neural ensembles in areas staged along the brain’s visual pathway. It is possible that the observed ANT modulations simply reflect propagating retinal signals. It is also possible that ANT modulations reflect extraretinally-mediated signals or a combination of both retinal and extraretinal signals as predicted by the active sensing model^25^. The early onset of fixation-locked modulation can be clearly distinguished from a visual evoked potential elicited by a visual stimulus. Specifically, we observed a positivity that began 260 ms and peaked 100 ms before fixation onset. Because median saccade duration which we observed was 27 ms, the onset of this ERP component is at least 233 ms before saccade onset, indicating that the ERP is not evoked by retinal movement due to saccades. This is also the case even if we consider some of the longest saccade durations (i.e., 80 ms).

Furthermore, the pre fixation ERP is followed by a negativity with the strongest deflection around 40 ms after fixation onset (i.e., around the time of BHA decrease). This is considerably faster than previously observed ANT responses to a visual stimulation. Štillová et al., observed that stimulus elicited evoked potentials started at about 100–150 ms with the strongest modulations detected about 250–300 and 700 ms after stimulus onset^51^. To further compare the time course of fixation- and stimulus-locked responses, we investigated modulations of field potentials locked to the onset of visual stimuli. This comparison was possible in one of the participants who viewed 480 images in a visual search task (160 unique pictures presented full screen and repeated 3 times). Based on prior studies, we hypothesized a later onset latency for stimulus-locked ERP. Directly comparing ERPs locked to fixation onset and stimulus onset, we note that although the overall morphology of the late ERP components was similar between fixation and stimulus locked responses, the pre-fixation positive and early negativity (N50) components were absent in the stimulus locked ERP. Also, ERP amplitude was greater in the stimulus-locked than in the fixation-locked condition. These observations dovetail with ITC and amplitude measures both of which show earlier response in fixation-locked condition, but overall greater amplitude in stimulus-locked condition (Fig. S1). Finally, we found that BHA increased steadily to peak around 400–500 ms after stimulus onset. Altogether, our results show that the neural activity in the ANT is modulated in different ways during free viewing and during passive visual stimulation.

### Distinct modulation of the ANT field potentials by different oculomotor events

We have shown above that neural activity in the ANT is modulated by both fixation (Fig. 1) and visual stimulus (Fig. S1) onset—each with a distinct time course. This finding adds a new dimension to our thinking about the role of ANT in cognition. These nuclei are usually considered in terms of their possible role in mnemonic rather the sensory or sensorimotor processes^38–40^. To further evaluate a possible ANT contribution to natural vision, we studied the influence of eye blinks on field potentials. The most imminent consequence of an eye blink is a temporary loss of visual input. Eye blinks differ from saccades in many ways (e.g., longer movement duration, lack of retinal input during movement and more variable movement duration), however like saccades, eye blinks are associated with extraretinal signals that modulate visual sensitivity^52–54^.

Studying the blink-offset field potentials, we observed a negative ERP deflection during the eye blink which peaked about 50–60 ms before the blink offset. It was followed by a positive deflection peaking about 100–140 ms after blink offset. We also found ITC exceeding the surrogate distribution across multiple time and frequency points with the strongest effects visible in frequencies below 10 Hz. Power modulation was weak to undetectable. Finally, the BHA showed a weak decrease during the blink which did not survive correction for multiple comparisons and a rebound that peaked about 200 ms after the blink offset which is similar to previous findings on the BHA modulations by the eye blinks observed in the cortex^53^.

Fixation-onset as well as visual stimulus-onset and blink-offset all reflect time points when the volley of retinal inputs is initiated in the retina and then processed by a succession of neural ensembles in areas staged along the brain’s visual pathway. There are important differences between ANT field potentials locked to these three events. First, ERPs locked to fixation-onset, stimulus-onset and blink-offset show different morphology. Eye blink offset-locked ERPs are transient and return to baseline right after the blink offset. This is in contrast to both fixation-and stimulus-locked ERPs both of which appear to oscillate. The ITC shows similar spectral content but distinct temporal profiles. The blink offset-locked ITC is more uniformly distributed before and after the eye event, while fixation- and stimulus-locked ITC are both strongest after the event. Finally, blink-offset locked BHA shows an increase peaking about 200 ms after the event which is similar to a previous study on blink-related potentials^53^. This is different from fixation-locked BHA, which shows a transient decrease right after fixation onset. Altogether, our results show that the ANT are engaged during natural vision and are differentially modulated by distinct visual and oculomotor events suggesting their possible role in natural vision.

## Discussion

To evaluate a possible role of the anterior nuclei of the thalamus (ANT) in natural vision, we studied neural dynamics surrounding fixations, visual stimulus onset and eye blinks during unconstrained free viewing, in three epilepsy patients implanted with ANT electrodes for clinical purposes. Comparing pre- and post-fixation measures to surrogate distributions, we found significant modulation of fixation-related field potential and related phase concentration (ITC), albeit with weak to undetectable concomitant changes in low frequency power. Fixation-related BHA, a measure of processes related to neuronal excitability^45–49^, showed a decrease from the surrogate distribution within the first 50 ms after fixation onset. This suggests that local neuronal excitability is weakly suppressed in the ANT right after fixation onset. Fixation-locked ERP displayed pre-fixation positivity that started 260 ms and peaked about 100 ms before fixation onset. It was followed by an early negative deflection beginning near fixation onset and peaking about 40–50 ms post-fixation followed by another positive deflection about 200 ms and another negativity at about 300 ms after fixation onset. Consistent with a previous report^51^, stimulus-locked ERP had an onset latency of 100–150 ms post-stimulus, followed by a negative deflection around 300 ms and late positivity around 600 ms after stimulus onset. Thus, the time course of fixation-locked ERP was different from the time course of stimulus-locked ERP. Overall our results of pre-fixation modulations of field potentials in the ANT are unlikely to be explained by fluctuation of retinal input due to the saccade. This is consistent with the view that in addition to their role in mnemonic processes, the ANT may play a role in natural vision.

Fixation-related phase concentration was strongest around the rate of saccades (3–4 Hz), peaking about 120 ms after fixation onset. The impact of saccades on oscillatory phase clustering in the ANT is consistent with findings from the MTL^16–24^ and the visual system^8–15^. However, the dynamics of fixation-related excitability modulation in the ANT, with a decrease in BHA around fixation onset, appears distinct from those reported for V1^9,11,15^. For example, Rajkai et al., observed the strongest suppression of neuronal firing just before and during saccades, but before the onset of fixation^9^, and essentially the same was observed by Barczak and colleagues^15^. Interestingly, dynamics similar to those in the ANT have been noted in higher order visual areas. For example, Zanos et al. observed a bimodal temporal distribution of suppression in V4 neurons, with suppression being strongest after fixation onset^14^. The decrease in BHA immediately after fixation onset coupled with strong increase in phase coherence suggests that the ANT may be reorganized to a low-excitability state immediately after saccades.

What role might the ANT play in visual active sensing? The anatomical connections of the ANT provide some suggestions. The ANT receive descending inputs from the subiculum and ascending inputs from the mammillary bodies forming a central component of the extended hippocampal–diencephalic system for episodic memory^40^. The ANT also have dense reciprocal connections to cingulate areas including the anterior cingulate cortex, which are implicated in top-down cognitive control (for review see^42^). Studies in nonhuman primates as well as tree shrews and rats^55,56^ have also identified direct input pathways from the retina and pretectum^57^ to the anterodorsal thalamus. Studies in nonhuman primates^43^ demonstrate that anterior thalamic nuclei have reciprocal connections to frontal and prefrontal areas, and through their intrathalamic connections, indirect access to posterior visual areas, placing the ANT within the circuits that could mediate top-down modulation due to attention and/or saccadic corollary signals. The combination of these diverse inputs from higher order brain regions suggest a possible role of the ANT in guidance and top-down control of sensing activities.

Functional properties of ANT neurons are also consistent with a role of ANT in the organization of sensing activities. In rodents, neurons in the ANT fire as a function of the animal’s head direction^36,58,59^, providing crucial input during spatial navigation by encoding animal’s perceived directional heading relative to its environment^60^. This is important to the present discussion, as rodents tend to favor head movements over saccades in active sensing. Although vestibular inputs are critical for generating head direction firing, the directional signal appears to also involve the motor system^60^. Taube and Burton showed that preferred direction firing is stable as the animal “actively” moves from one environment to another^61^. However, “passive” transportation of an animal between two environments distorts directional firing and this distortion seems to be independent of the visual input^60,62^. Overall, the interconnectivity patterns of the ANT together with the functional properties of ANT neurons suggest that the ANT contributes more to top-down control, than to bottom-up input processing in active sensing.

How does mediation of control during natural vision fit with other functions that have been attributed to the ANT? The ANT have been proposed to contribute to a range of cognitive functions including learning and memory^38–40^ as well as attention^63–65^. Damage to the ANT or its inputs from the mammillary bodies leads to episodic memory deficit observed in Wernicke-Korsakoff and thalamic stroke patients^66^. Interestingly, along with other cognitive deficits, both Wernicke-Korsakoff and thalamic stroke patients have difficulty in motor sequence programming^67,68^. This last may be a key observation, because overt active sensing depends on motor sampling routines—sequences of movements by the eyes, whiskers, head, hands and breathing musculature. In an active sensing framework, these motor sequences imbue individual’s sampling strategy with momentary predictions and allow transcription of the sensory world into a set of neural representations that the brain can use to generate perception and to guide further adaptive behavioral routines^25,31–33,69^.

What other thalamic regions might contribute to the signals recorded within the ANT and what do we know about their functions? For the analysis in this study we re-referenced our recordings forming bipolar montages. The bipolar derivation increases spatial specificity of field potentials by subtracting signals shared across neighboring channels. Because the ANT is relatively small^70^ even with the bipolar montage we might be measuring signals over an area exceeding a single nucleus which is a possible explanation for a reversed polarity on the left shaft in patient 2. The neighboring nuclei that might have contributed to our recordings include mediodorsal nucleus (MD) and internal medullary lamina^41^. The MD with its reciprocal connections with the frontal lobe including prefrontal cortex and anterior cingulate cortex has been shown to play a role in multiple cognitive functions (for review see^71^) including both episodic^38^ and working memory^72,73^. The MD is an important node in an ascending pathway from the superior colliculus to the frontal eye field that conveys corollary discharge signal including information on upcoming eye movement^30,74^. Research in nonhuman primates showed that inactivating MD impairs motor tasks that require monitoring of eye movements consistent with loss of corollary discharge^74^. Studies in humans with lesions to the MD showed impairments to both motor and visual updating^75,76^. While less is known about the role of internal medullary lamina (IML), previous research in cats suggest that parts of the IML are involved in the mechanism of gaze orientation^77^.

There are several reasons why it is unlikely that the current results reflect extraocular muscle or facial muscle artifacts rather than genuine neural modulations of the ANT. First, in the fixation-locked field potential, the strongest effects we found were those that follow fixation onset, while eye movement artifacts should be strongest during the saccade^78,79^. Second, typical extraocular muscle artifacts manifest as a broadband power increase across spectrum^78,79^ centered at the movement onset with distinct spectral properties resulting from different sources. This is opposite to what we have found—weak to undetected power in low frequencies (< 30 Hz), with concomitant increase in the ITC and BHA amplitude decrease. Third, like extraocular muscle artifacts, face muscle activity may also contaminate intracranial recordings and manifest as an increase in broadband amplitude (see a case report^80^). As noted above, we observed weak to undetectable change in low frequency power (< 30 Hz) and a decrease in BHA which is an opposite pattern to that expected from facial and extraocular muscle activity^80^. For all these reasons, it is unlikely that the current results reflect extraocular or facial muscle artifacts.

The current study cannot identify the precise circuits underlying the early fixation-locked field potential modulations. The connectivity profile of the ANT which receives both retinal afferents and inputs from higher order cortices is consistent with the idea that fixation-related modulation reflects contributions from both early non-retinal signals and later retinal inputs. To experimentally separate retinal and non-retinal signals one would need to record ANT activity in the dark or match visual input in passive and active visual search. While we were unable to implement these tests in the patients reported here, the fact that the earliest deflection in the fixation-locked ERPs precede the saccade onset timing by at least 200 ms argues that the ANT receive substantial modulation from saccade-related signals that are nonretinal in origin. This finding suggests that in addition to their functions in episodic memory, the ANT likely play a role in natural active vision.

### Potential limitations of this study

We analyzed data from only three patients. However, this in itself is not a critical limitation, as we have a large number of repeated measures within the individual patients (fixations: N = 1019, 820, 637) and our results are visible at multiple ANT channels and reproduced in each participant. A more obvious limitation is that while EOG-based eye movement detection provides great temporal precision and high accuracy in detecting large saccades comparable with modern eye tracking^50,81^, it is less sensitive to small eye movements. It is therefore possible that the current study underestimates the contribution of shorter saccades (and microsaccades) and over-estimates fixation durations. To ensure that our results are robust across fixation durations, we performed control analyses in which we limited maximum duration of fixation to 600 and 800 ms resulting in distributions with median durations centered at 300 ms or 500 ms (comparable to those known in real life visual exploration; e.g.,^82^). Both of these control analyses showed similar results—significant ERP deflection, increased ITC, decreased BHA and weak to undetected effects on power. The EOG-based eye tracking provides little information about the direction of gaze. Thus, while reporting saccadic modulation of ANT activity, our study leaves open the question of whether gaze direction and foveal content are systematically reflected in the field potentials of ANT activity. We used Fp1 referenced to linked mastoids for detecting vertical part of EOG in patients 1–3 and bipolar EOG 2–EOG1 in patients 1 and 3 and F7–F8 in patient 2 for detecting horizontal EOG. EOG signals have been shown to spread across all these channels with varying magnitude^50^. Despite these slight differences in electrode placement, we find consistent effects and similar eye movement distributions across all three patients, confirming that our results are robust across these variations in electrode position. Overall, there is no reason to think these limitations have a major influence on our main conclusions.

Previous studies recorded eye movements in the dark to separate stimulus evoked and saccade related signals. Although our findings do not identify precise origins of these signals they rule out retinal causes for the early modulation components; i.e., those that precede saccade onset. The earliest ERP component we found was detectable about 260 ms before fixation onset. Given a median saccade duration of 27 ms, this time point is at least 233 ms before saccade onset. Because the modulation starts during the preceding fixation, it is unlikely to be solely explained by saccade induced changes in retinal input. Altogether, the current findings that the neural activity in the ANT is modulated by extra-retinal influence represents a significant contribution to our understanding of the ANT functions. In addition to their role in episodic memory, the current results suggest that the ANT play a role in guiding or regulating saccadic sampling of the environment during natural vision.

## Methods

### Participants

Depth field potentials along with the surface EEG were recorded from 3 pharmacoresistant epilepsy patients (age range from 22 to 52, 2 males and 1 female) implanted with electrodes targeted to the anterior nuclei of the thalamus for treatment of epilepsy. Recordings were performed at the Department of Epileptology, University of Bonn, Germany. The study was approved by the Ethics Committee of the University of Bonn Medical Center. The study was performed in accordance with the relevant guidelines and regulations and all patients gave written informed consent.

### Electrophysiological recordings

The data were recorded using bilaterally implanted multi-contact (4 channels per shaft) depth electrodes and simultaneous scalp surface EEG electrodes (Fp1, Fp2, F3, F4, C3, C4, 'P3, P4, O1, O2, F7, F8, T3, T4, T5, T6, Cz, Fz, Pz, T1, T2, Cb1, Cb2) in patient 2 and additionally EOG1, EOG2 in patients 1 and 3. Surface electrodes were placed according to the 10–20 system. Note that in the current study we only used surface electrodes to detect EOG signal. To that end, we considered channels closest to the eye orbits. Other electrodes were recorded for research unrelated to the subject of this manuscript. The depth electrodes’ contacts (Medtronic 3387) were of 1.5 mm height with an inter-contact center-to-center spacing of 3 mm. All data were sampled at 1 kHz, on-line band pass filtered between 0.01 Hz (6 dB/octave) and 300 Hz (6 dB/octave), off-line down-sampled to 500 Hz. The ANT depth channels were re-referenced to its neighbor using bipolar montage to increase local specificity of the signal^83^. This results in 3 bipolar pairs per subject per side. In total we analyzed 18 bipolar pairs (3 pairs on shaft × 2 sides × 3 subjects). The data consist of continuous 1 h long unconstrained recordings during which participants performed various cognitive tasks involving presentation of visual stimuli on a laptop computer, interacted with experimentators and members of the hospital staff.

### Electrode localization

Surgical procedures followed these described previously^41^. Briefly, the locations of contacts were estimated relative to midcommissural point (MCP) using the AC-PC coordinate system and relative to visible landmarks such as mamillothalamic tract (MTT) ANT junction (MTT/ANT junction). The procedure utilizes both visible anatomical landmarks in individual patient MRIs and stereotactic atlas information. The most relevant anatomical landmark is the MTT that joins ANT nucleus at its inferior border slightly anterior to the midpoint of ANT in the anterior–posterior axis.

Preoperative navigation sequences were acquired with a 3.0-T MR imaging unit (Achieva, Philips Healthcare, Best, the Netherlands) and included high-spatial-resolution three-dimensional T1-weighted sequences pre- and postcontrast (isotropic three-dimensional gradient echo; voxel size, 1.0 × 1.0 × 1.0 mm; echo time, 3.48 ms; repetition time, 7.53 ms). Correct electrode placement was verified by postoperative high-spatial-resolution CT (Brilliance 16, Philips Healthcare, Best, the Netherlands; voxel size, 0.8 × 0.8 × 0.8 mm).

The electrodes were first mapped onto brain using co-registration by iELVis^84^ followed by electrode identification on post-implantation CT co-registered to pre-implantation T1 image. To obtain the anatomical location labels presented in Fig. 1, we used Freesurfer’s automated segmentation^85^.

**Figure 3:**
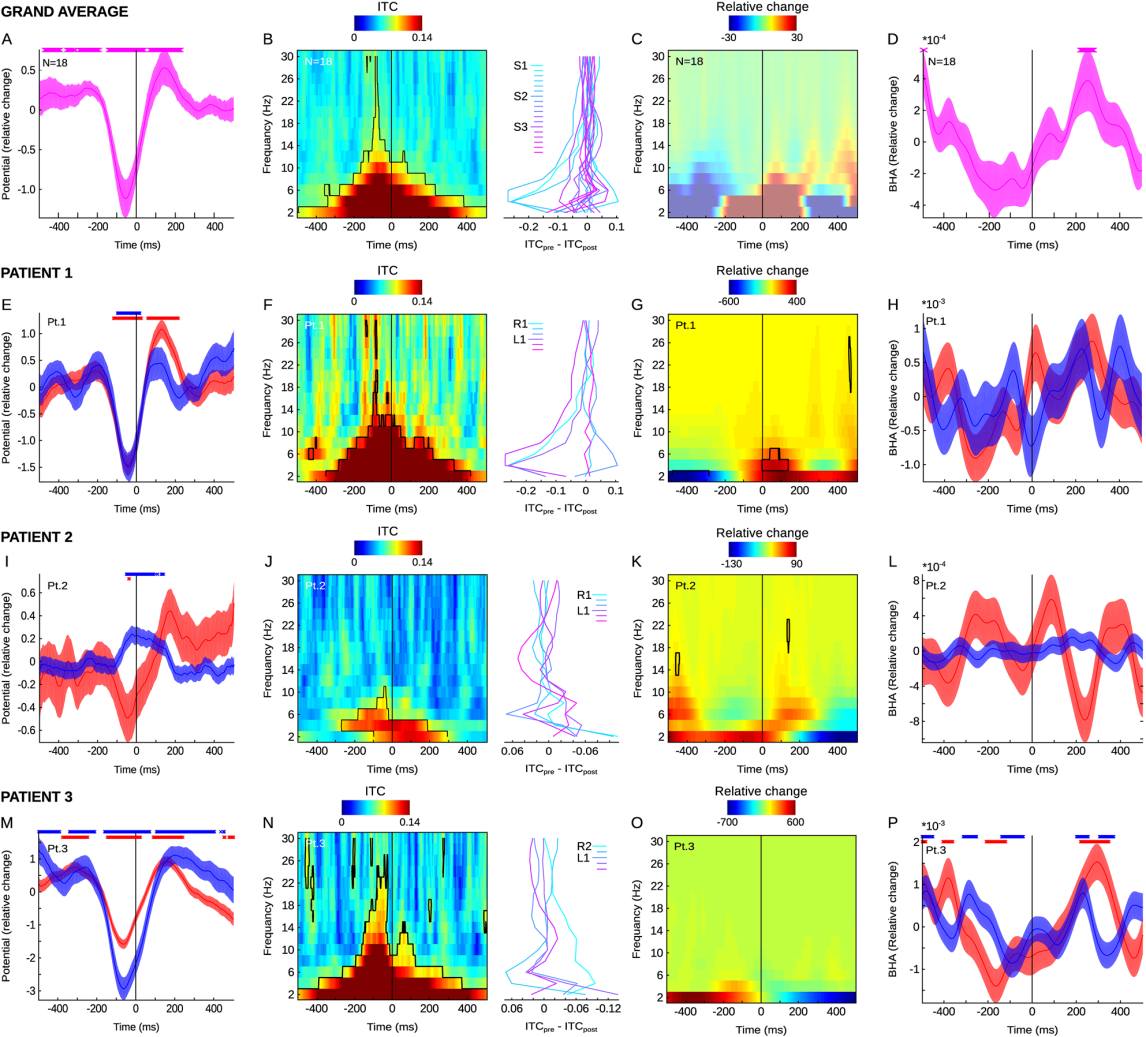
Blink offset-locked neural activity in human ANT. (**A**) Grand average for blink offset-locked field potentials (N = 18 ANT channels, 3 patients). Vertical lines show the point of blink offset (i.e., onset of visual input). Markers above indicate significant time points (permutation test *p* < 0.05). (**B**) Color map shows ITC (time on x-axis, frequency on y-axis). Contours depict significant time–frequency points (permutation test *p* < 0.05). Vertical black line indicates blink offset (i.e., the beginning of visual input). Line plots on the right side indicate ITC change from pre- (− 300: − 100 ms) to post- blink offset (0:100 ms) time window separately for each channel. (**C**) Color map shows blink offset locked power relative to trial average (time on x-axis, frequency on y-axis). No change from surrogate distribution (permutation test *p* < 0.05). (**D**) Blink offset locked BHA (80–180 Hz). Vertical black line indicates blink offset (i.e., beginning of visual input). Markers above indicate significant time points (permutation test *p* < 0.05). (**E**) Blink offset-locked field potentials (patient 1) from channels on the right ANT (blue) and left ANT (red). Vertical black lines show the point of blink offset (i.e., beginning of visual input). Markers above indicate significant time points (permutation test *p* < 0.05). (**F**) Same as B but data from patient 1. (**G**) Same as C but data from patient 1. Contours present change from surrogate distribution (permutation test *p* < 0.05) (**H**) Blink offset-locked BHA (patient 1) on the right ANT (blue) and left ANT (red). Vertical black lines show the point of blink offset. Data from patient 2 (**I**–**L**) and patient 3 (**M**–**P**) plotted with the same convention as (**E**–**H**). All results are controlled for multiple comparisons with Benjamini and Hochberg/Yekutieli false discovery rate procedure. Shading reflects standard error of the mean (SEM)﻿.

In the current study patient 3 had the right side electrode placed deep with ventral channels showing no signal (note only R2 and R3 are presented in Figs. 2 and 3). Furthermore, careful inspection of ERPs on the left and right ANT shaft show that while right side ERPs in patient 2 are consistent with other patients, the ERPs recorded from the left shaft show reversed ERP polarity (see Fig. 2).

### Data analyses

All data were processed offline using MATLAB (MathWorks). Pre-processing was performed at single electrode level. Continuous data were down-sampled to 500 Hz. To remove line noise, we used a butterworth filter (4th order) at 50 Hz, 100 Hz, 150 Hz. Next, we created bipolar montage to maximize spatial specificity of the signal and also to decrease common noise and contributions from distant sources through volume conduction. To this end, we subtracted signals from neighboring contacts on the electrode (i.e., ANTR0–ANTR1, ANTR1–ANTR2, ANTR2–ANTR3 for right side contacts and ANTL0–ANTL1, ANTL1–ANTL2, ANTL2–ANTL3 for left side contacts, respectively). ANTR0 is the deepest contact on the right shaft. Low frequency complex-valued activity (1–30 Hz) was extracted from continuous data with 3 cycles wavelets. The phase and power was extracted from this complex-valued signal (for similar approach see^86–90^).

Broadband high frequency activity (BHA) was calculated for frequencies 80–180 Hz in 5 Hz steps using sliding Hanning tapered window (150 ms) with 6 Hz spectral smoothing. The complex-valued signal was rectified and squared to extract power and averaged across frequencies to create a single vector of BHA fluctuations (for similar approach see^49,91–93^).

Next, we used scalp channels to create continuous signal maximizing vertical and horizontal components of the Electrooculogram (EOG). The EOG is a technique for measuring corneo-retinal standing potential that exists between the front and the back of the human eye. Several previous reports established the feasibility of measuring eye movements with this method. We acknowledge the limited spatial specificity of the EOG-based eye tacking in the limitations section and withhold from any statements regarding gaze location or direction of movement. At the same time EOG has excellent (i.e., comparable to modern eye tracking temporal resolution which makes it suitable for the purpose of detecting time points of fixations, saccades and eye blinks (e.g.,^50,81^). This is important because despite the rich and naturalistic environment in which the study was conducted, it allowed us to precisely control the critical condition in the current study (i.e., the time points of fixations and eye blinks). It enabled us to anchor our analyses to the end points of eye movements and compute fixation-locked neural signals, a method that is well established by previous studies^9,11,13–15,23,49,94^.

Here, we used an Fp1 channel (referenced to linked mastoids) for the vertical component in all three patients and bipolar EOG2-EOG1 for patients 1 and 3 as well as F7-F8 for patient 2. Selection was based on channel availability per patient and known scalp distribution of the EOG gradient^50^. To define fixation onsets, we used a probabilistic algorithm for detection of EOG events^81^ which has been shown to achieve very high sensitivity and precision for detecting saccades and fixations—over 96% for saccade larger than 4.3 degrees of visual angle and over 90% for saccades larger or than 2.9 degrees of the visual angle. We used an initial 20% of data (i.e., 12 min) for an unsupervised training period which is required by the algorithm. The eye events were then detected in the remaining 80% of data (48 min) which entered further analyses. We detected 1959, 1868 and 1635 number of saccades for patients 1–3, respectively.

To sort blinks from saccades, we used a probabilistic algorithm for detecting blinks, saccades, and fixations^81^. Briefly, there are two features of the EOG signal which are used by the classifier developed by Toivanen et al. to tell apart these three eye events. First, for fixations, the derivative of the filtered horizontal and vertical EOG is close to zero as produced by a steady signal. In turn, during saccades and eye blinks the value of the derivative is high. This feature is useful for separating fixations from blinks and saccades. To further separate fixations from eye blinks the algorithm considers a second feature which is based only on the derivative of the vertical EOG signal. The feature is defined as the difference between successive maxima and minima of the derivative, subtracted with the absolute value of the sum of these: Dv = max − min − |max + min|. The reason for this feature is that a blink produces a distinctively symmetrical pattern in the derivative of the vertical signal and should thus have a higher value for Dv than a saccade (for more details see Toivanen et al., 2015).

Continuous BHA, complex-valued time–frequency signal and raw field potentials were segmented into 400 ms long non-overlapping windows relative to each fixation with 200 ms before and 200 ms after each event. For further analyses we only considered fixations that were not preceded or followed by another fixation within the +/− 200 ms window of interest. Based on previous studies we further constrained fixation durations to last below 800 ms^82^. We reasoned that longer fixations were likely spurious due to intervening small saccades that were undetected with the current setup. This resulted in 1019, 820, 637 epochs per patient 1–3, respectively. We defined epochs containing artifacts as those with gradient of field potential exceeded 5 standard deviation of the trial mean. On average (across patients) this criterion marked about 3% of epochs as containing artifacts. These segments were removed from further analyses. We calculated ITC, based on fixation-locked complex-valued signal using previously described formula^95^ with the following Matlab implementation:

$${\text{ITC}} = {\text{abs}}\left( {{\text{mean}}\left( {{\text{exp}}\left( {1{\text{i}}*{\text{angle}}\left( {{\text{complex - valued}}\;{\text{signal}}} \right)} \right)} \right.} \right.$$

We tested whether fixation-locked field potentials, BHA, ITC or power in frequencies 1–30 Hz differed from what would be expected by chance. To test this, we created surrogate distributions (N = 1000 permutations) for each of the signals of interest (i.e., ERP, BHA. ITC, power). The surrogate distributions were created by locking intact segments of ANT signals to pseudo-events (i.e., random time points uniformly distributed across the entire recording session). For each permutation we randomly selected “pseudo-events”—these were random time points from our 1-h long recording session which matched the number of eye events. Next, we created surrogate data segments by extracting 400 ms time epochs around each “pseudo-event”. We used the same procedure of artifact rejection to remove artifacts from our surrogate distribution as we did from our empirical data. Next, each empirical signal (fixation-onset locked ERP, ITC, BHA, power) was tested against its surrogate distribution with significance threshold set to 5th and 95th percentile (corresponding to *p* < 0.05). We used False Discovery Rate (FDR) correction for multiple comparisons^96^ across time (ERP, BHA) and time–frequency (ITC).

We reasoned that to test whether observed modulations to our signals of interest (i.e., saccade-/blink offset-locked spectral ITC, power, ERP and BHA) are stronger than chance, it is sufficient to disrupt the relation between field potentials and eye movements (by locking our surrogate analyses to randomly selected time points). This should break any synchronization between eye movements and spectral ITC, power, ERP and BHA.

While breaking this relation constitutes a sufficient test, surrogates, unlike our empirical event-locked data, might possibly contain saccades before and/or after the pseudo-event. Because there is no systematic relation of the eye movement across measurements in our surrogate data, this should not bias our surrogate distribution. However, we directly tested this posibility with a control analysis (Fig. S2) in which we ensured there is no eye movement or blink either before or after a “pseudo-event” similarly to our empirical data. Briefly, with these alternative surrogate distributions, we reproduced all our results—(1) increased ITC; (2) ERP modulation as well as (3) decrease in the BHA (see Fig. S2). While the effects of power differ between surrogates, we noted above that power modulations appeared largely unreliable across individual channels and at best showed weak to undetected effects.

### Stimulus-locked analyses

To study the time course of neural response elicited by visual stimuli (Fig. S1), we analyzed field potentials locked to stimulus onset. The precise timing of stimulus onset was only available for one subject (patient 1). Briefly, the patient performed a visual search task. The total of 480 images were presented (i.e., 160 unique images each presented three times). Each picture was displayed until response for the maximum duration of 3 s followed by a uniformly gray screen presented for the random duration ranging from 100 to 500 ms with the median duration of 250 ms. The task was to search for a yellow mark (with about 50% transparency to increase difficulty) hidden in the image. The participant indicated whether the mark was displayed on the right or left side of an image. Unlike our main analyses, here we anchored field potentials to the stimulus onset. Based on a previous study^51^, we expected that the earliest latency of visual evoked potential should be about 115–150 ms with the maximum response amplitude around 300 ms after stimulus onset. We used the same approach to extract BHA, ITC and power as described above. Because the time point of stimulus onset was preceded by a clearly defined baseline interval during which a gray screen was presented we quantified responses relative to the baseline period. To this end, we compared the magnitude of post-stimulus signal intensity (ERP, BHA, power) to the baseline using the Wilcoxon sign rank test. We also used Rayleigh test for non-uniformity of circular data to quantify phase clustering. We used False Discovery Rate (FDR) correction for multiple comparisons^96^ across time (ERP, BHA) and time–frequency (ITC, power).

## Supplementary Information

Supplementary Figures.
