# Supplementary material for: Neural activity in the human anterior thalamus during natural vision
Source: Sci Rep. 2021 Sep 1;11:17480. doi: 10.1038/s41598-021-96588-x (PMC8410783; doi:10.1038/s41598-021-96588-x)
Supplement: Supplementary file 1 — Supplementary Figures. [file 41598_2021_96588_MOESM1_ESM.docx]

**Supplementary material**

**Neural activity in the human anterior thalamus**

**during natural vision**

Marcin Leszczynski^1,2*^, Leila Chaieb^3^, Tobias Staudigl^4^, Simon Jonas Enkirch^5^,

Juergen Fell^3^, Charles E. Schroeder^1,2^

**^*^**Correspondence to

Dr. Marcin Leszczynski

Department of Psychiatry,

Columbia University College of Physicians and Surgeons,

1051 Riverside Drive Kolb Annex Rm 561

New York, NY 10032

Email: [leszczynski.marcin@gmail.com](mailto:leszczynski.marcin@gmail.com)


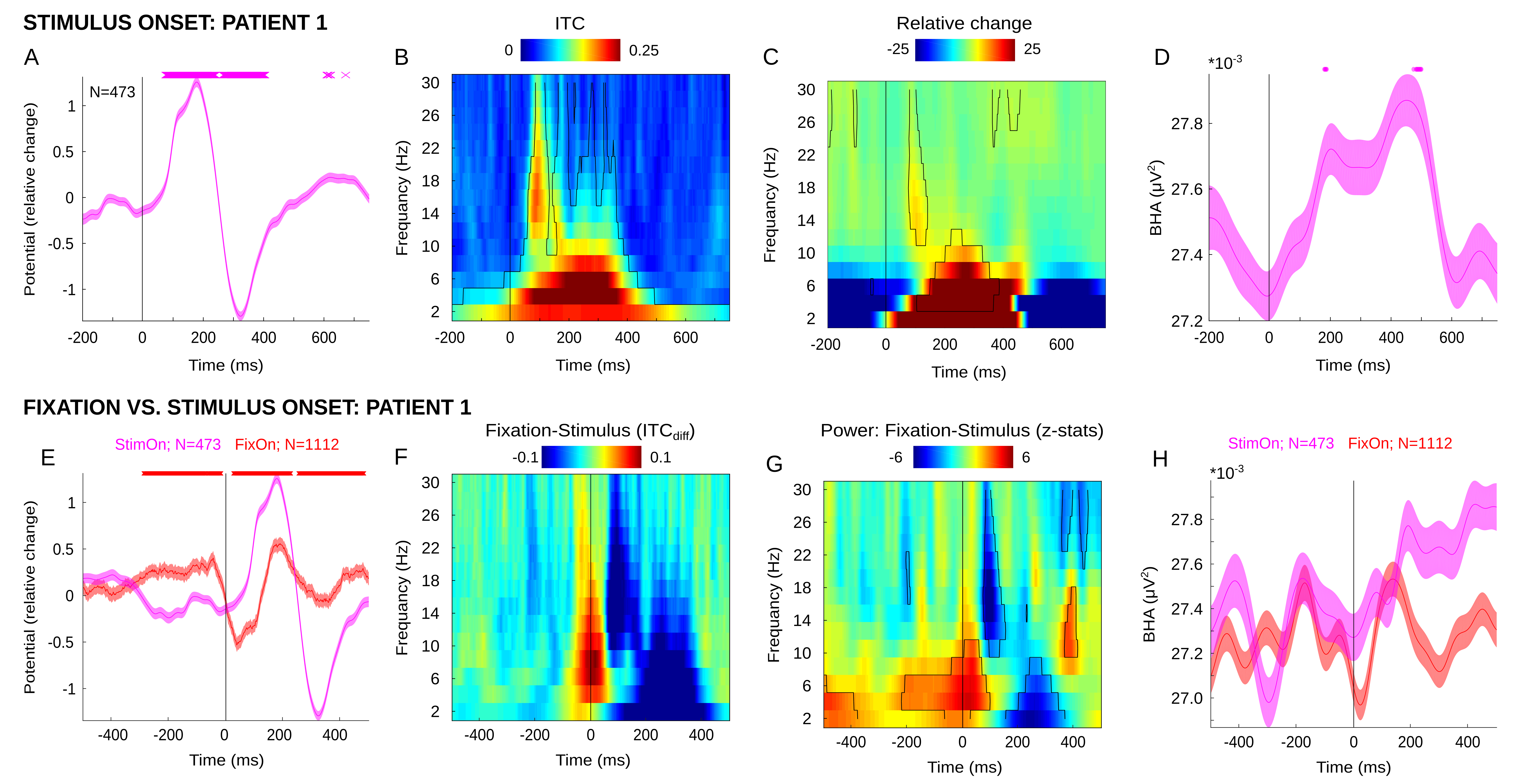
***Figure S1. Visual stimulus onset*** ***locked neural activity in the human ANT (patient 1).*** ***(A)*** *Average stimulus onset-locked field potentials normalized to trial average (N = 473 colored natural images presented full screen; data from patient 1). Vertical line shows the point of stimulus onset. Markers above indicate significant time points (p < 0.05 relative to 100 ms pre-stimulus baseline; Wilcoxon sign rank test).* ***(B)*** *Color map shows inter-trial phase coherence (time on x-axis, frequency on y-axis). Contours depict significant time-frequency points (**p < 0.05; Rayleigh test). Vertical black line indicates stimulus onset.* ***(C)*** *Shows stimulus onset locked power normalized to trial average (time on x-axis, frequency on y-axis). Contours depict significant time-frequency points relative to prestimulus baseline (p < 0.05; Wilcoxon sign rank test).* ***(D)*** *Stimulus onset locked BHA. Vertical black line indicates stimulus onset. Markers above indicate significant time points (p < 0.05; Wilcoxon sign rank test uncorrected).* ***(E)*** *Average stimulus onset-locked field potentials (magenta) and fixation onset-locked potential (red). Vertical line shows the point of stimulus/fixation onset. Markers above indicate significant difference between stimulus and fixation ERPs (p < 0.05; Wilcoxon rank sum test).* ***(F)*** Color map shows time-frequency difference between fixation-locked and stimulus onset locked ITC. Vertical line shows the point of stimulus/fixation onset. Data is from a single patient. Unlike comparisons in E, G, H in which trials served as repeated measures, ITC is a measure across repetitions. **(G)** Color map shows time-frequency z-statistics (*Wilcoxon rank sum test*) result from a comparison between fixation-locked and stimulus onset locked power. Vertical line shows the point of stimulus/fixation onset. Contours present significant time-frequency points controlled for multiple comparisons (p<0.05). **(H)** *Average stimulus onset-locked BHA (magenta) and fixation onset-locked BHA (red). Vertical line shows the point of stimulus/fixation onset. Unless stated differently all results are controlled for multiple comparisons with Benjamini & Hochberg/Yekutieli false discovery rate procedure. Shading reflects standard error of the mean (SEM).*


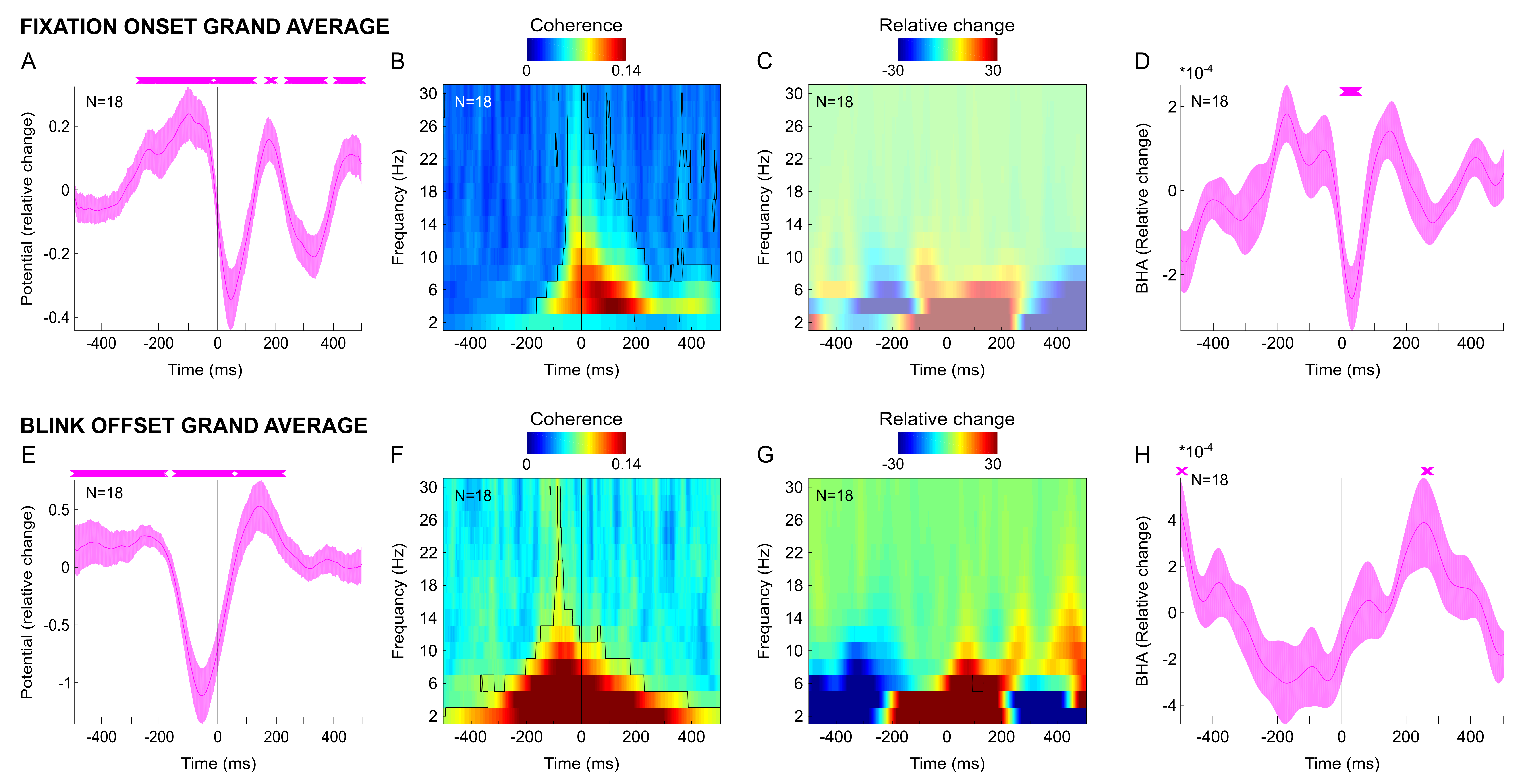
**Figure S2.** ***Fixation onset locked and blink offset locked neural activity in human ANT.*** *We reasoned that to test whether observed modulations to our signals of interest (i.e., saccade-/blink offset-locked spectral ITC, power, ERP and BHA) are stronger than chance, it is sufficient to disrupt the relation between field potentials and eye movements (by locking our surrogate analyses to randomly selected time points). This should break any synchronization between eye movements and spectral ITC, power, ERP and BHA. While breaking this relation constitutes a sufficient test, surrogates, unlike our empirical event-locked data, might possibly contain saccades before and/or after the pseudo-event. Because there is no systematic relation of the eye movement across measurements in our surrogate data, this should not bias our surrogate distribution. This figure shows results of a control analysis in which the surrogate distributions were created by locking intact segments of ANT signals to pseudo-events (i.e., random time points uniformly distributed across the entire recording session) similarly to that in Fig 2 and 3 in the main manuscript. Additionally, any eye event within +/-200ms window of interest was excluded from the surrogate data. Independently of the way we create surrogates, we found reliable modulations of ERP, ITC, BHA and weak to undetected modulations to power (<30 Hz).* ***(A)*** *Grand average fixation-locked field potentials (N = 18 ANT channels, 3 patients). Vertical lines show the point of fixation onset. Markers above indicate significant time points (p < 0.05; permutation test).* ***(B)*** *Color map shows ITC (time on x-axis, frequency on y-axis). Contours depict significant time-frequency points (permutation test p < 0.05). Vertical black line indicates fixation onset.* ***(C)*** *Color map shows fixation locked power relative to trial average (time on x-axis, frequency on y-axis). No significant point was detected.* ***(D)*** *Fixation-locked BHA (80-180Hz). Vertical black line indicates fixation onset. Markers above indicate significant time points (permutation test p < 0.05).* ***(E-H)*** *Same as A-D but data is locked to blink offset. All results are controlled for multiple comparisons with Benjamini & Hochberg/Yekutieli false discovery rate procedure. Shading reflects standard error of the mean (SEM).*
